# The prognostic performance of the log odds of positive lymph nodes in patients with esophageal squamous cell carcinoma: A population study of the US SEER database and a Chinese single‐institution cohort

**DOI:** 10.1002/cam4.4120

**Published:** 2021-07-09

**Authors:** Hongdian Zhang, Wanyi Xiao, Peng Ren, Kai Zhu, Ran Jia, Yueyang Yang, Lei Gong, Zhentao Yu, Peng Tang

**Affiliations:** ^1^ Department of Esophageal Cancer Tianjin Medical University Cancer Institute and Hospital Key Laboratory of Cancer Prevention and Therapy of Tianjin Tianjin's Clinical Research Center for Cancer National Clinical Research Center of Cancer Tianjin China; ^2^ Department of Thoracic Surgery National Cancer Center National Clinical Research Center for Cancer Cancer Hospital & Shenzhen Hospital Chinese Academy of Medical Sciences and PeKing Union Medical College Shenzhen China

**Keywords:** a Chinese single‐institution cohort, esophageal squamous cell carcinoma, log odds of positive lymph nodes, prognosis, SEER database

## Abstract

**Background:**

The purpose of this study was to assess the prognostic performance of the log odds of positive lymph nodes (LODDS) value compared with the pathological N stage and lymph node ratio (LNR) in patients with esophageal squamous cell carcinoma (ESCC).

**Method:**

In total 1144 patients diagnosed with ESCC from the Surveillance, Epidemiology, and End Results (SEER) database and 930 patients from our validation cohort were eligible. Kaplan–Meier plotter and multivariate Cox proportional hazards models were conducted to investigate the prognostic value of the N stage, LNR stage, and LODDS stage. The homogeneity, discriminatory ability, and monotonicity of these variables were evaluated using the linear trend *χ* ^2^ test, likelihood ratio *χ* ^2^ test, Akaike information criterion (AIC), and consistency index (C‐index) to determine the potential superiorities.

**Results:**

The prognostic LODDS cutoff values were determined to be −1.49 and −0.55 (*p* < 0.001). Univariate analyses showed significant association among the N, LNR, and LODDS stages and overall survival of the patients (all *p* < 0.001). Multivariate analyses confirmed that the LODDS stage remained an independent prognostic indicator in both the SEER database and our validation cohort. Subgroup analyses identified the ability of LODDS stage to distinguish heterogeneous patients within various groups in both independent databases. Furthermore, the model with the highest C‐index and smallest AIC value was the one incorporating the LODDS stage among the three investigated nodal classifications of both cohorts.

**Conclusion:**

The novel LODDS stage demonstrated better prognostic performance than the traditional N or LNR stages in ESCC patients. It can serve as an auxiliary factor to improve prognostic performance and can be applied to evaluate the lymph node status to increase the precision of staging and evaluation of survival.

## Graphical abstract

The LODDS classification showed a better prognostic performance than the traditional N or LNR stages in patients with ESCC. LODDS can serve as an auxiliary factor to improve prognostic performance and can be applied in the evaluation of lymph node status.

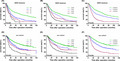

## 1 INTRODUCTION

Esophageal cancer is a common malignant tumor of the gastrointestinal tract, and surgical resection remains the mainstay treatment for patients diagnosed with resectable esophageal cancer.^1^ However, with the continuous improvement of surgical techniques and application of multidisciplinary comprehensive treatment therapies, the overall 5‐year survival rate remains very low.^2^, ^3^ Lymph node metastasis (LNM) has been proven to be an important prognostic factor in esophageal cancer patients.^4^ Therefore, to achieve a better prognosis, a precise lymph node (LN) staging system should be developed to optimize postoperative treatment and follow‐up.

In 2017, the eighth edition tumor–node–metastasis (TNM) staging system by the International Union Against Cancer (UICC)/American Joint Committee on Cancer (AJCC) proposed an updated pathologic N stage on the basis of the number of positive LNs.^5^ However, the absolute number of positive LNs is restricted by the total number of removed LNs, and insufficient removed LNs may lead to stage migration.^6^ The recently reported optimal numbers of removed LNs for esophageal squamous cell carcinoma (ESCC) vary widely, with medians ranging from 6 to 30.^7^

Thus, multiple studies have made efforts to address this problem and proposed many auxiliary measurements to aid the TNM staging system. The lymph node ratio (LNR), referring to the ratio of the number of metastatic LNs to the total number of removed LNs, can more accurately reflect the degree of tumor burden from the perspective of definition and somehow reduce stage migration.^8^ The prognostic role of the LNR remains controversial, because some researchers advocate its superiority over the N stage,^9^, ^10^ while others have questioned the correlation between the LNR and survival rate when all or no LNs exhibit metastasis.^11^, ^12^ The LNR cannot efficiently identify prognostic heterogeneity among patients.

The log odds of positive lymph nodes (LODDS),^13^ defined as the logarithm of the ratio between the number of metastatic LNs and number of negative lymph nodes (NLNs), has been proven to be a predictive power of prognosis and has shown superiority to other LN status‐based assessments in various cancers.^10^, ^11^, ^14^, ^15^, ^16^ The cutoff points vary from different studies in different tumors, and it is difficult to reach an agreement because of the limited number of reports.^17^, ^18^, ^19^ However, little clinical evidence exists regarding the prognostic impact of the LODDS on ESCC prognosis and the comprehensive regimen to incorporate multiple clinical factors for prognostication.

Therefore, this study was aimed to elucidate the value of the LODDS classification in predicting the long‐term survival of ESCC patients using the population‐based Surveillance, Epidemiology, and End Results (SEER) database and to further validate this finding in a single Chinese institution.

## 2 MATERIALS AND METHODS

### 2.1 Data source and patients

This was a retrospective analysis involving two different databases to compare the ability of three LN classifications to predict survival. The clinical and pathological data of patients with ESCC from 2004 to 2015 were obtained from the open‐access SEER database, a large population‐based cancer database that collects cancer incidence data from 18 cancer registries. The selection criteria for screening patients were as follows: (1) histological type confirmed as primary ESCC; (2) aged older than 18 years; (3) underwent an esophagectomy; (4) dissection of at least two LNs; and (5) active follow‐up information. Patients who met the following criteria were excluded: (1) adoption of neoadjuvant therapy before surgery; (2) distant metastasis; (3) history of prior malignancy; or (4) incomplete clinical pathology data. Approval for this study from the Institutional Review Board was exempt because SEER is a publicly available database.

Another independent single‐institution Chinese cohort from Tianjin Medical University Cancer Institute and Hospital comprising 930 patients who underwent an Ivor Lewis or Meckown transthoracic esophagectomy with LN dissection for ESCC between 2004 and June 2015 was developed using the same inclusion and exclusion criteria, and was used as the validation dataset.

We retrieved the baseline and clinical characteristics, including patient demographics (age, gender, and race), tumor characteristics (tumor location and tumor size), pathological characteristics (histological type, total number of removed LNs, T stage, and N stage), and follow‐up information. Notably, the baseline characteristics of the two groups were not identical, as our cohort dataset lacks the data of race, but contains additional information about smoking history and body mass index (BMI) compared with the SEER dataset.^20^ The Ethical Committee and Institutional Review Board of Tianjin Medical University Cancer Institute and Hospital granted ethical approvals.

We defined overall survival (OS) as the time interval from the date of diagnosis to the last follow‐up time or death due to any cause for survival analysis.

### 2.2 LN classification definition

Based on the eighth edition of the TNM staging system, the N category was classified based on the number of metastatic LNs: N0, no metastasis; N1, 1–2 LNs; N2, 3–6 LNs; and N3, ≥7 LNs. The LNR interval was defined as the metastatic LNs counts divided by the total number of removed LNs. The LODDS value was calculated using the formula log [(pN + 0.5)/(nN + 0.5)], where pN is the number of positive LNs and nN is the number of NLNs removed.

### 2.3 Statistical analysis

All statistical analyses and plotting graphics were performed using SPSS 26.0 software (IBM Corp) and the R 4.0.2 (R Foundation for Statistical Computing) statistical package. For all statistical analyses, *p* < 0.05 was identified as statistically significant.

The descriptive statistics were presented as the median [interquartile range (IQR)] for continuous variables that met the normal distribution, whereas counts and percentages were presented for categorical variables. The correlations among the LNM, LNR, and LODDS were visualized by scatter plots, and the degree of linear relationships on the scatter plots was evaluated by Spearman's correlation analysis (*r*). The X‐tile program (https://medicine.yale.edu/lab/rimm/research/software.aspx), a better way to access the prognostic value of continuity indexes, was employed to determine the optimal cutoff points for the LNR and LODDS stages based on minimal probability (*P*) values.^21^

The OS curves of patients were depicted by Kaplan–Meier survival analysis, and the log‐rank test was used to elucidate the differences between groups. Univariate analysis was first performed to identify potential prognostic factors among candidate variables. The statistically significant variables were then integrated into the multivariate Cox regression analysis to obtain independent prognostic risk factors. Second, we separately incorporated the N stage (Model 1), LNR stage (Model 2), and LODDS stage (Model 3) to establish three predictive Cox regression models. Third, the above three stages were integrated with other potential predictors to create the fourth Cox regression model (Model 4). Hazard ratios (HRs) and 95% confidence intervals (CIs) were calculated. Nomograms were constructed to provide a visualized risk prediction method based on the risk factors verified by multivariate analyses using the survival and rms packages of R software.

The prognostic performance of the aforementioned different LN models was compared in terms of multiple dimensions, including monotonicity, homogeneity, and discrimination. A higher linear trend *χ* ^2^ score showed better discriminatory ability and monotonicity of each model. The higher the likelihood ratio *χ* ^2^ score, the better the homogeneity for each model. Furthermore, the goodness of fit for each model was evaluated by the Akaike information criterion (AIC). A lower AIC often indicated a better model fit.

Harrell's concordance index (C‐index) was calculated to assess the predictive capacity and fit of these aforementioned predictive models with values ranging from 0.5 to 1. Previous studies have suggested that a C‐index ranging from 0.50–0.70 indicates a low degree of distinction, a moderate degree of distinction between the interval 0.70 and 0.90, and a high degree of distinction higher than 0.90.^22^ Stratification analyses visualized by tree diagrams were used to evaluate the performance of the LODDS classification in distinguishing heterogeneous patients within various groups according to the results of the multivariate analyses.

## 3 RESULTS

### 3.1 Clinical and pathological characteristics of the patients

After selection, 1144 patients from the US SEER database and 930 patients from the Chinese cohort met the eligibility criteria and were included in this study. The detailed patient information of the two cohorts is presented in Table 1. The enrolled patients comprised 711 males and 433 females with a median age of 65 (IQR, 58, 72) years in the SEER cohort. In our validation cohort, there were 770 males and 160 females with a median age of 61 (IQR, 55, 68) years. Additionally, 684 (59.8%) and 409 (44.0%) patients had fewer than 16 LNs removed in the SEER cohort and our validation cohort, respectively. Regarding patients with more than 16 removed LNs, 460 (40.2%) and 521 (56.0%) cases were in the two datasets, respectively.

**TABLE 1:** Clinical and pathological characteristics of patients with ESCC in SEER database and our cohort

	No. of patients (percent)	
	SEER database	Our cohort
Year of diagnosis (year)		
2004–2009	620 (54.2%)	453 (48.7%)
2010–2015	524 (45.8%)	477 (51.3%)
Age (year)		
Median (IQR)	65 (58, 72)	61 (55, 68)
<65	584 (51.0%)	576 (61.9%)
≥65	560 (49.0%)	354 (38.1%)
Gender		
Male	711 (62.2%)	770 (82.8%)
Female	433 (37.8%)	160 (17.2%)
Race		
White	186 (16.3%)	NA
Black	821 (71.8%)	NA
Others	137 (12.0%)	NA
Smoking history		
None	NA	298 (32.0%)
Yes	NA	632 (68.0%)
Tumor location		
Upper	90 (7.9%)	60 (6.5%)
Middle	515 (45.0%)	555 (59.7%)
Lower	539 (47.1%)	315 (33.9%)
Histological type		
G1	86 (7.5%)	39 (4.2%)
G2	582 (50.9%)	706 (75.9%)
G3	476 (41.6%)	185 (19.9%)
Tumor size (mm)		
Median (IQR)	40.5 (27, 70)	40 (30, 50)
<45	657 (57.4%)	594 (63.9%)
≥45	487 (42.6%)	336 (36.1%)
BMI		
1	NA	494 (53.1%)
2	NA	373 (40.1%)
3	NA	63 (6.8%)
T stage		
T1	257 (22.5%)	79 (8.5%)
T2	215 (18.8%)	199 (21.4%)
T3	603 (52.7%)	377 (40.5%)
T4a	69 (6.0%)	275 (29.6%)
N stage		
N0	777 (67.9%)	499 (53.7%)
N1	268 (23.4%)	271 (29.1%)
N2	74 (6.5%)	110 (11.8%)
N3	25 (2.2%)	50 (5.4%)
LNR stage		
LNR0	777 (67.9%)	499 (53.7%)
LNR1	85 (7.4%)	117 (12.6%)
LNR2	115 (10.1%)	169 (18.2%)
LNR3	167 (14.6%)	145 (15.6%)
LODDS stage		
LODDS1	412 (36.0%)	294 (31.6%)
LODDS2	556 (48.6%)	512 (55.1%)
LODDS3	176 (15.4%)	124 (13.3%)
Removed lymph nodes		
<16	684 (59.8%)	409 (44.0%)
≥16	460 (40.2%)	521 (56.0%)

Abbreviations: BMI, body mass index; ESCC, esophageal squamous cell carcinoma; IQR, interquartile range; LNR, lymph nodes ratio; LODDS, log odds of positive lymph nodes; SEER, Surveillance, Epidemiology, and End Results database.

In the SEER cohort, the median follow‐up time was 32 months and the 5‐year OS rate was 37.2%. Moreover, the median follow‐up time was 49.1 months with a 5‐year OS rate of 45.1% in our validation cohort.

### 3.2 Distribution of LNM, LNR, and LODDS

**FIGURE 1:**
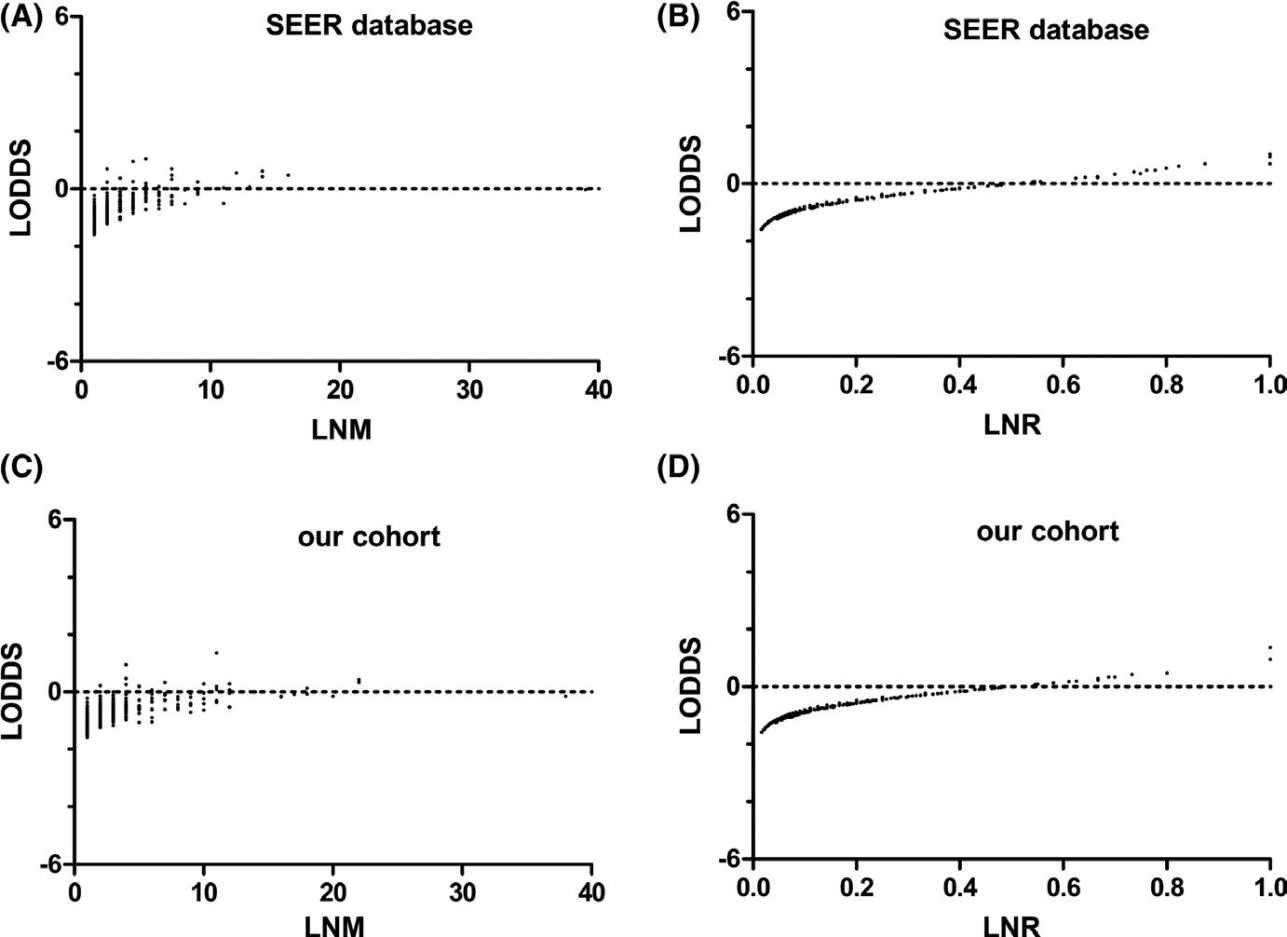
The distribution of LODDS and LNM (A, *r* = 0.690), LODDS and LNR (B, *r* = 0.721) in the SEER database; LODDS and LNM (A, *r* = 0.846), LODDS and LNR (B, *r* = 0.884) in our validation cohort (Spearman's rank test). LODDS, log odds of positive lymph nodes; LNR, lymph node ratio; LNM, number of lymph node metastases

We created scatter plots to comprehensively and reasonably assess the correlations among the three different LN classifications. As depicted in Figure 1, the LODDS values increased with increases in the number of metastatic LNs and the LNR. However, the coefficient of correlation between LODDS and the LNR was higher than that between LODDS and the number of metastatic LNs in the SEER database (*r* = 0.721 vs. *r* = 0.690) and our validation cohort (*r* = 0.884 vs. *r* = 0.846), implying that the correlation between LODDS and the LNR was more prominent.

### 3.3 Identification of the cutoff values and characteristics of nodal classification

The N stage was classified as follows: in the SEER database, 777 (67.9%) cases were N0, 268 (23.4%) cases were N1, 74 (6.5%) cases were N2, and 25 (2.2%) cases were N3. Consistent with the X‐tile analysis of the SEER cohort, the optimal cutoff points for the LNR were 0.06 and 0.16. The distribution was as follows: 777 (67.9%) cases in the LNR0 group (LNR = 0), 85 (7.4%) cases in the LNR1 group (LNR < 0.06), 115 (10.1%) cases in the LNR2 group (LNR, 0.06–0.16), and 167 (14.6%) cases in the LNR3 group (LNR, 0.16–1). The cases were separated into three groups according to the LODDS cutoffs based on X‐tile software: 412 (36.0%) cases were enrolled in the LODDS1 group (LODDS < −1.49), 556 (48.6%) cases were enrolled in the LODDS2 group (LODDS, −1.49 to −0.55), and 176 (15.4%) cases were enrolled in the LODDS3 group (LODDS > −0.55).

Correspondingly, in our validation cohort, the N stage was classified as follows: N0 (*n* = 499), N1 (*n* = 271), N2 (*n* = 110), and N3 (*n* = 50). LNR was classified as follows: LNR0 (*n* = 499), LNR1 (*n* = 117), LNR2 (*n* = 169), and LNR3 (*n* = 145). The LODDS scheme was determined using the aforementioned interval values: LODDS1, 294 (31.6%); LODDS2, 512 (55.1%); and LODDS3, 124 (13.3%).

**FIGURE 2:**
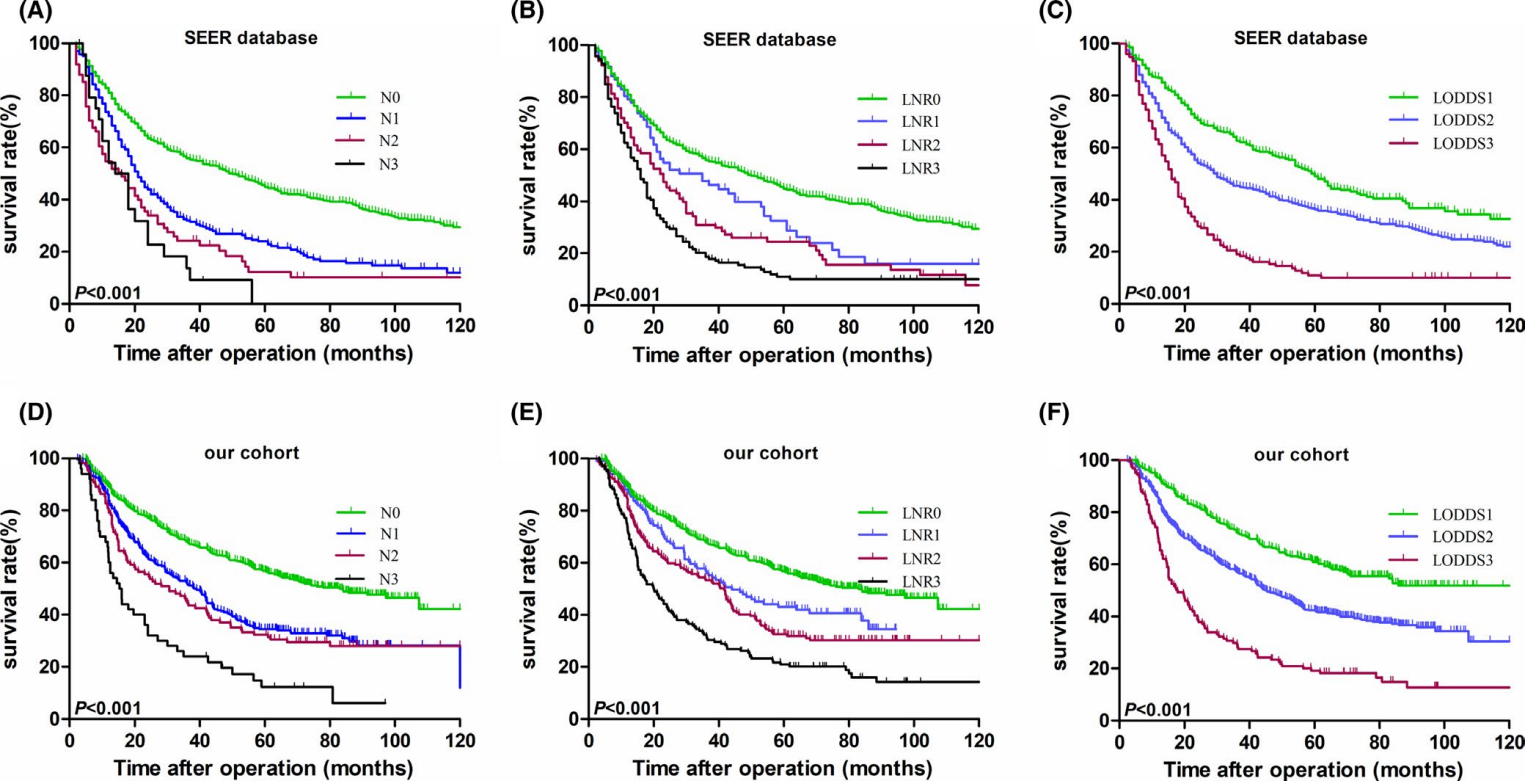
Kaplan–Meier curves for the 5‐year OS stratified by N stage (A), LNR stage (B), and LODDS stage (C) for the SEER database and by N stage (D), LNR stage (E), and LODDS stage (F) for our validation cohort

The Kaplan–Meier survival curves for the two cohorts stratified by all three variables are presented in Figure 2. The differences in survival between subgroups for the N stage, LNR stage, and LODDS stage were statistically significant (all *p* < 0.001, Figure 2A–C) in the SEER cohort. Identical results were also obtained (all *p* < 0.001, Figure 2D–F) for our validation cohort.

### 3.4 Univariate and multivariate survival analyses

Table 2 summarizes the detailed results of the univariate and multivariate analyses of the SEER database. All the factors, including the year of diagnosis, age, gender, race, tumor location, histological type, tumor size, T stage, N stage, LNR stage, and LODDS stage, were significantly associated with OS in the univariate survival analyses. Then, these risk factors, except for LN‐associated variables were used to establish a model as a basis for the next four‐step multivariate analysis. Subsequently, N stage was embedded into the first step of multivariate analysis. The results indicated that the involved variables (*p* < 0.05 for all) were all independent risk factors. After that, the LNR instead of N stage was incorporated into this model to conduct the second multivariate analysis and was also confirmed to be significant (*p* < 0.001). In the third multivariate analysis, the LODDS was incorporated, and the results were similar to those of the second multivariate analysis: the LODDS was confirmed to be significant with other variables. Prognostic nomograms based on the N stage, LNR stage, and LODDS stage were developed from the three final models (Figure S1). In the last step, the three LN categories were all added into the model of step 4 multivariate analysis to see the difference. Notably, N and LNR were found to be inferior to LODDS and became statistically nonsignificant themselves (*p* = 0.659 and 0.656, respectively).

**TABLE 2:** Univariable and multivariable Cox regression analyses of prognostic factors in the SEER database

Characteristics	Univariable analysis		Multivariable analysis 1		Multivariable analysis 2		Multivariable analysis 3		Multivariable analysis 4	
	HR (95% CI)	*P*	HR (95% CI)	*P*	HR (95% CI)	*P*	HR (95% CI)	*P*	HR (95% CI)	*P*
Year of diagnosis (year)		**0.004**		**0.013**		**<0.001**		**0.047**		**0.038**
2004–2009	Reference		Reference		Reference		Reference		Reference	
2010–2015	0.782 (0.663–0.923)		0.809 (0.684–0.956)		0.817 (0.691–0.965)		0.846 (0.715–0.992)		0.837 (0.707–0.990)	
Age (year)		**0.022**		**<0.001**		**<0.001**		**<0.001**		**<0.001**
<65	Reference		Reference		Reference		Reference		Reference	
≥65	1.190 (1.025–1.381)		1.390 (1.189–1.625)		1.390 (1.189–1.624)		1.383 (1.183–1.616)		1.387 (1.187–1.622)	
Gender		**0.008**		0.056		**0.044**		**0.031**		**0.047**
Male	Reference		Reference		Reference		Reference		Reference	
Female	0.809 (0.691–0.947)		0.855 (0.728–1.004)		0.847 (0.721–0.995)		0.837 (0.713–0.984)		0.849 (0.722–0.997)	
Race		**0.013**		**0.009**		**0.018**		**0.033**		**0.031**
White	Reference		Reference		Reference		Reference		Reference	
Black	0.749 (0.618–0.908)		0.729 (0.595–0.894)		0.746 (0.609–0.915)		0.776 (0.633–0.950)		0.768 (0.626–0.943)	
Others	0.823 (0.624–1.084)		0.821 (0.618–1.089)		0.832 (0.628–1.104)		0.918 (0.691–1.220)		0.895 (0.672–1.191)	
Tumor location		**0.006**		**0.001**		**0.002**		**0.001**		**0.001**
Upper	Reference		Reference		Reference		Reference		Reference	
Middle	0.769 (0.589–1.003)		0.748 (0.570–0.983)		0.775 (0.590–1.016)		0.776 (0.591–1.018)		0.760 (0.578–1.000)	
Lower	0.661 (0.506–0.865)		0.616 (0.467–0.812)		0.634 (0.482–0.835)		0.630 (0.479–0.829)		0.615 (0.466–0.811)	
Histological type		**<0.001**		**0.011**		**0.012**		**0.017**		**0.020**
G1	Reference		Reference		Reference		Reference		Reference	
G2	1.887 (1.332–2.673)		1.492 (1.046–2.128)		1.457 (1.022–2.078)		1.441 (1.011–2.055)		1.446 (1.013–2.064)	
G3	2.228 (1.571–3.159)		1.684 (1.178–2.407)		1.662 (1.163–2.373)		1.628 (1.140–2.325)		1.623 (1.135–2.321)	
Tumor size (mm)		**0.002**		**0.041**		**0.042**		**0.037**		**0.039**
<45	Reference		Reference		Reference		Reference		Reference	
≥45	1.263 (1.087–1.468)		1.181 (1.007–1.385)		1.179 (1.006–1.383)		1.183 (1.010–1.387)		1.182 (1.008–1.386)	
T stage		**<0.001**		**<0.001**		**<0.001**		**<0.001**		**<0.001**
T1	Reference		Reference		Reference		Reference		Reference	
T2	1.366 (1.057–1.765)		1.319 (1.015–1.712)		1.317 (1.014–1.711)		1.341 (1.032–1.741)		1.328 (1.022–1.726)	
T3	2.034 (1.659–2.496)		1.824 (1.470–2.263)		1.820 (1.467–2.257)		1.897 (1.532–2.350)		1.838 (1.481–2.280)	
T4a	3.016 (2.202–4.130)		2.550 (1.842–3.530)		2.539 (1.837–3.509)		2.698 (1.957–3.721)		2.611 (1.886–3.614)	
N stage		**<0.001**		**<0.001**						0.659
N0	Reference		Reference						Reference	
N1	1.766 (1.488–2.096)		1.542 (1.295–1.838)						1.184 (0.514–2.729)	
N2	2.479 (1.888–3.254)		2.149 (1.622–2.847)						1.420 (0.620–3.250)	
N3	3.029 (1.967–4.663)		2.008 (1.288–3.131)						1.309 (0.531–3.225)	
LNR stage		**<0.001**				**<0.001**				0.656
LNR0	Reference				Reference				Reference	
LNR1	1.385 (1.047–1.831)				1.268 (0.956–1.682)				0.949 (0.400–2.255)	
LNR2	1.842 (1.456–2.331)				1.606 (1.265–2.039)				1.110 (0.506–2.431)	
LNR3	2.458 (2.021–2.988)				2.004 (1.635–2.456)					
LODDS stage		**<0.001**						**<0.001**		**0.002**
LODDS1	Reference						Reference		Reference	
LODDS2	1.540 (1.290–1.839)						1.503 (1.255–1.800)		1.400 (1.154–1.699)	
LODDS3	2.898 (2.327–3.610)						2.379 (1.895–2.986)		1.871 (0.836–4.190)	

Abbreviations: SEER, Surveillance, Epidemiology, and End Results database; HR, hazard ratio; CI, confidence interval; LNR, lymph nodes ratio; LODDS, log odds of positive lymph nodes.

Bold indicates statistically significant values (*p* < 0.05).

The results of the survival analyses of the Chinese validation cohort are presented in Table 3. In the univariate analyses, the year of diagnosis, age, smoking history, histological type, tumor size, T stage, N stage, LNR stage, and LODDS stage were statistically significant (*p* < 0.05). The results of the N‐, LNR‐, and LODDS‐based Cox multivariate analyses were assessed in accordance with the abovementioned steps for the SEER database. N stage, LNR stage, and LODDS stage combined with the year of diagnosis, age, tumor size, and T stage were independent prognostic factors in the first, second, and third multivariate analyses. The corresponding prognostic nomograms of the three models are shown in Figure S2. Finally, the fourth multivariate analysis incorporated all three LN classifications. In accordance with the results of the SEER database, the LODDS stage was still an independent prognostic predictor, while the N stage and LNR stage were nonsignificant (*p* = 0.309 and 0.359, respectively).

**TABLE 3:** Univariable and multivariable Cox regression analyses of prognostic factors in our cohort

Characteristics	Univariable analysis		Multivariable analysis 1		Multivariable analysis 2		Multivariable analysis 3		Multivariable analysis 4	
	HR (95% CI)	*P*	HR (95% CI)	*P*	HR (95% CI)	*P*	HR (95% CI)	*P*	HR (95% CI)	*P*
Year of diagnosis (year)		**<0.001**		**0.003**		**0.006**		**0.041**		**0.036**
2004–2009	Reference		Reference		Reference		Reference		Reference	
2010–2015	0.727 (0.614–0.861)		0.760 (0.635–0.910)		0.777 (0.649–0.930)		0.829 (0.692–0.993)		0.822 (0.684–0.987)	
Age (year)		**0.008**		**0.043**		**0.029**		**0.025**		**0.032**
< 65	Reference		Reference		Reference		Reference		Reference	
≥65	1.257 (1.061–1.491)		1.202 (1.006–1.438)		1.220 (1.020–1.460)		1.227 (1.026–1.467)		1.216 (1.017–1.455)	
Gender		0.133								
Male	Reference									
Female	0.838 (0.665–1.056)									
Smoking history		**0.043**		0.063		0.060		0.053		0.059
None	Reference		Reference		Reference		Reference		Reference	
Yes	1.210 (1.006–1.456)		1.205 (0.990–1.466)		1.206 (0.992–1.465)		1.213 (0.997–1.474)		1.208 (0.993–1.470)	
Tumor location		0.271								
Upper	Reference									
Middle	1.276 (0.873–1.866)									
Lower	1.370 (0.927–2.026)									
Histological type		**0.012**		0.072		0.098		0.151		0.122
G1	Reference		Reference		Reference		Reference		Reference	
G2	2.051 (1.204–3.493)		1.719 (0.999–2.958)		1.663 (0.966–2.863)		1.622 (0.942–2.792)		1.649 (0.958–2.839)	
G3	2.310 (1.326–4.026)		1.931 (1.093–3.413)		1.855 (1.049–3.281)		1.759 (0.994–3.112)		1.810 (1.023–3.202)	
Tumor size (cm)		**<0.001**		**0.036**		**0.018**		**0.012**		**0.020**
<45	Reference		Reference		Reference		Reference		Reference	
≥45	1.456 (1.227–1.727)		1.218 (1.013–1.464)		1.248 (1.039–1.498)		1.263 (1.053–1.515)		1.244 (1.035–1.495)	
BMI		**0.007**		0.137		0.079		0.071		0.085
1	Reference		Reference		Reference		Reference		Reference	
2	0.855 (0.712–1.028)		0.908 (0.754–1.094)		0.892 (0.740–1.076)		0.864 (0.716–1.041)		0.870 (0.721–1.049)	
3	1.424 (1.036–1.958)		1.264 (0.913–1.750)		1.295 (0.936–1.791)		1.234 (0.893–1.704)		1.231 (0.890–1.705)	
T stage		**<0.001**		**0.005**		**0.010**		**0.002**		**0.002**
T1	Reference		Reference		Reference		Reference		Reference	
T2	1.733 (1.134–2.650)		1.184 (0.764–1.836)		1.186 (0.765–1.838)		1.169 (0.755–1.811)		1.144 (0.738–1.773)	
T3	2.449 (1.644–3.648)		1.429 (0.941–2.170)		1.403 (0.923–2.131)		1.448 (0.955–2.195)		1.421 (0.936–2.157)	
T4a	2.916 (1.946–4.372)		1.764 (1.151–2.703)		1.716 (1.119–2.631)		1.813 (1.185–2.773)		1.789 (1.167–2.742)	
N stage		**<0.001**		**<0.001**						0.309
N0	Reference		Reference						Reference	
N1	1.728 (1.425–2.096)		1.578 (1.291–1.927)						1.223 (0.635–2.357)	
N2	1.922 (1.489–2.482)		1.695 (1.291–2.226)						1.056 (0.588–1.898)	
N3	3.633 (2.629–5.021)		3.105 (2.226–4.332)						1.554 (0.784–3.082)	
LNR stage		**<0.001**				**<0.001**				0.359
LNR0	Reference				Reference				Reference	
LNR1	1.405 (1.075–1.836)				1.298 (0.985–1.712)				0.874 (0.446–1.712)	
LNR2	1.770 (1.418–2.210)				1.591 (1.260–2.009)				1.105 (0.594–2.056)	
LNR3	2.749 (2.204–3.429)				2.419 (1.922–3.045)					
LODDS stage		**<0.001**						**<0.001**		**0.006**
LODDS1	Reference						Reference		Reference	
LODDS2	1.687 (1.374–2.071)						1.580 (1.276–1.956)		1.420 (1.095–1.841)	
LODDS3	3.460 (2.668–4.489)						3.159 (2.406–4.148)		2.506 (1.289–4.875)	

Abbreviations: BMI, body mass index; CI, confidence interval; HR, hazard ratio; LNR, lymph nodes ratio; LODDS, log odds of positive lymph nodes.

Bold indicates statistically significant values (*p* < 0.05).

### 3.5 Subgroup analyses stratified by different characteristics

**FIGURE 3:**
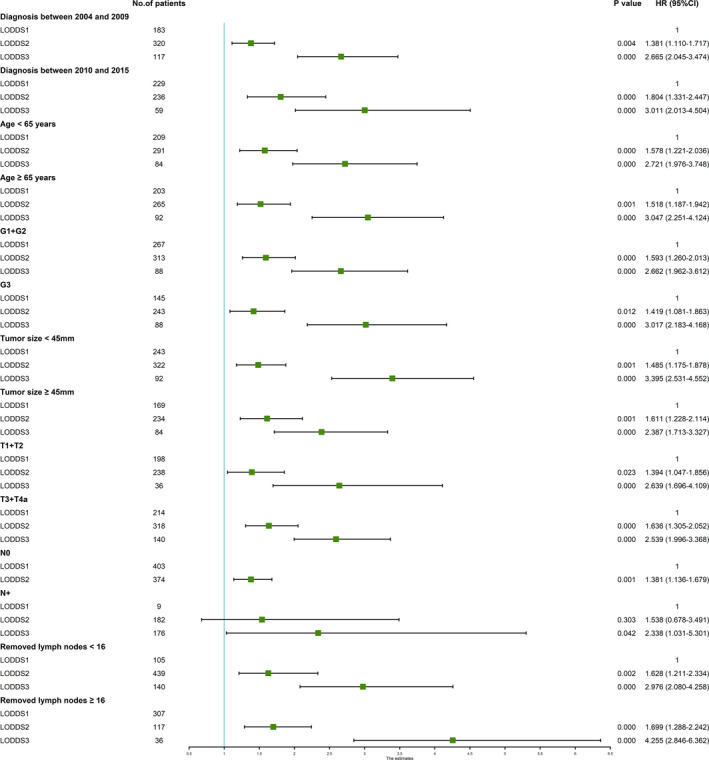
Forest plot depicting subgroup analysis results of the multivariate Cox regression model stratified by various risk factors for the 5‐year survival of the SEER database

**FIGURE 4:**
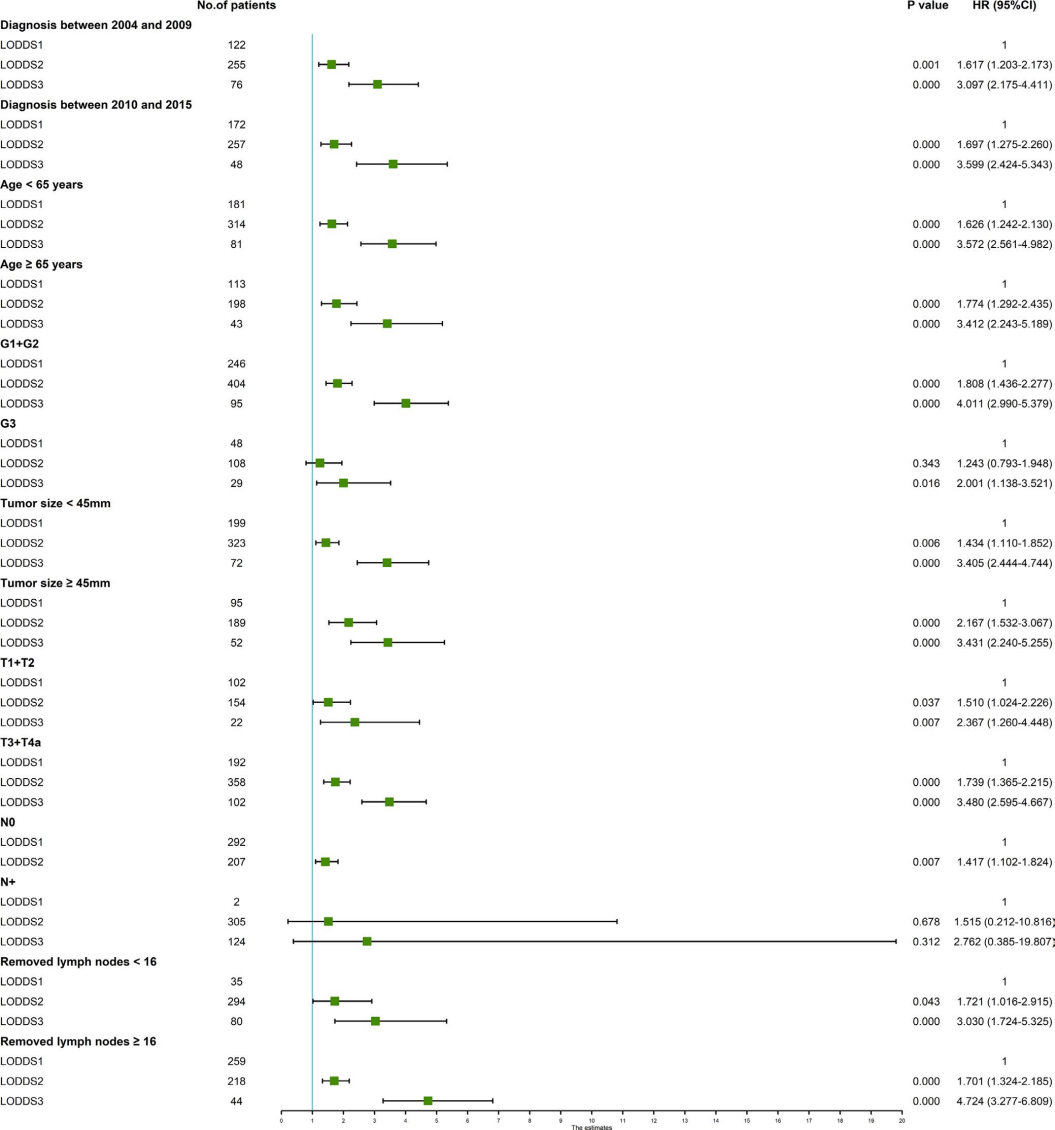
Forest plot depicting subgroup analysis results of the multivariate Cox regression model stratified by various risk factors for the 5‐year survival of our validation cohort

To explore the performance of the LODDS classification in predicting the OS of patients, we conducted subgroup analyses stratified by different characteristics in a multivariate Cox regression model based on the data retrieved from the two cohorts. As presented in Figure 3, the LODDS classification remained a prognostic predictor in ESCC patients regardless of the year of diagnosis (between 2004 and 2009 or between 2010 and 2015), age (<65 years or ≥65 years), histological type (G1 + G2 or G3), tumor size (<45 mm or ≥45 mm), T stage (T1 + T2 or T3 + T4a), LNM status (N0 or N+), and number of removed LNs (<16 or ≥16) in the SEER database. Similar results were observed in the analysis of our Chinese validation dataset. However, LODDS could not stratify LN‐positive patients with a significant difference (Figure 4).

### 3.6 Comparison of the discriminative ability of prognostic prediction models containing different LN classifications

To further evaluate the predictive capacity and accuracy of these node‐based models, linear trend *χ* ^2^, likelihood ratio *χ* ^2^, and AIC values were calculated (Table 4). We found that the linear trend *χ* ^2^ and likelihood ratio *χ* ^2^ values of the LODDS stage were higher than those of the N stage and LNR stage in both cohorts. The AIC values were 8724.66, 8719.17, and 8717.29 for the N, LNR, and LODDS stages, respectively, in the SEER database. Regarding the validation cohort, the AIC values were 6920.40, 6910.79, and 6907.01 for the N, LNR, and LODDS stages, respectively.

**TABLE 4:** Prognostic ability and accuracy of different lymph node staging system

Classification	Subgroups	SEER database				Our cohort			
		Linear trend *χ* ^2^	Likelihood ratio *χ* ^2^	AIC	C‐index	Linear trend *χ* ^2^	Likelihood ratio *χ* ^2^	AIC	C‐index
N Stage	N0, N1, N2, and N3	50.53	77.37	8724.66	0.659	49.58	68.61	6920.40	0.647
LNR Stage	LNR0, LNR1, LNR2, and LNR3	56.49	82.87	8719.17	0.664	59.89	78.22	6910.79	0.649
LODDS Stage	LODDS1, LODDS2, and LODDS3	80.65	84.75	8717.29	0.671	59.94	82.01	6907.01	0.658

Abbreviations: AIC, Akaike information criterion; C‐index, concordance index; LNR, lymph node ratio; LODDS, log odds of positive lymph nodes.

Furthermore, the C‐indexes for predicting OS among the N‐, LNR‐, and LODDS‐based nomograms were 0.659, 0.664, and 0.671, respectively, in the SEER dataset. For the validation cohort, the C‐indexes were 0.647 (N stage), 0.649 (LNR stage), and 0.658 (LODDS stage).

## 4 DISCUSSION

In our study, retrospective statistics of the SEER database showed that the LODDS stage was a robust risk factor for prognostic prediction, and this finding was then confirmed in our Chinese validation dataset. The developed Cox regression model identified the LODDS stage as a useful index superior to traditional N stage and LNR stage in predicting the OS of ESCC patients after curative operation.

Esophageal cancer is a particularly aggressive gastrointestinal malignancy, and its incidence and LNM rates are higher than those of other digestive cancers.^23^ The LNM status of ESCC patients is recognized as one of the critical determinants of prognosis after surgical treatment and is crucial for accurate staging; this system may prevent stage migration and allow individualized treatment.^24^ Among the LN classifications that have been proposed, the traditional and widely used eighth edition N staging system is not sufficient to comprehensively assess the LNM status because the number of negative and removed LNs, a critical factor to estimate prognosis, are not considered.^10^

Considering this situation, several studies have adopted novel LN classification systems combined with the number of removed LNs to assess ESCC prognosis. To the best of our knowledge, the number of NLNs,^25^, ^26^ ratio between negative and positive LNs (R_NP_),^27^ LNR,^28^ and LODDS^29^ are promising prognostic risk variables among several alternative systems.^30^

The LNR has shown superiority over the N stage in reflecting the degree of LNM and predicting patient survival according to some previous studies.^8^, ^31^ A previous survival analysis of 387 ESCC patients from a single‐center cohort found that the LNR classification and the proposed tumor–ratio–metastasis (TRM) stage showed superiority to the conventional N stage and TNM stage in predicting OS.^28^ However, the correlation between the LNR and survival rate merits further investigation, particularly when all or no LNs exhibit metastasis.^32^

To date, the LODDS value has been proposed as a novel prognostic indicator that is more accurate than existing indexes in patients with various cancers.^33^, ^34^ However, its accuracy in ESCC has yet to be explored.^13^, ^15^ The LODDS stage system has potential superiority because it considers both the information of the involved nodes and the NLNs. The singularity due to null observations is avoided by adding 0.5 to the numerator and denominator.

Given its reflection on both the number of negative and positive LNs situation, the LODDS classification was revealed to have prognostic value in esophageal cancer and demonstrated to have better performance than the number of positive LNs and LNR in a study of 1667 ESCC patients with pT3 stage.^15^ Considering this enlightening finding, we would like to investigate the prognostic performance of the LODDS stage compared with that of other node schemes and further validate the results in a large cohort, providing evidence to describe the LODDS stage as a sensitive staging system. This was the first study based on a large number of ESCC patients to assess LODDS stage using X‐tile software for cutoff optimization. Furthermore, we also used a Chinese single‐institution cohort to verify whether the determined LODDS classification remained significant in predicting prognosis, and the results were positive.

The prognostic power of different LN stages was confirmed in the univariate analyses of the SEER database analysis. The 5‐year OS rates decreased markedly with increasing LNR and LODDS subgroup values. Multistep multivariate analyses demonstrated that either the N, LNR, or LODDS was an independent prognostic risk factor. However, when all three variables were introduced into one model as covariates, the LODDS stage remained statistically significant, but not the N stage or LNR stage. Additionally, this phenomenon was observed in our validation cohort, suggesting that the prognostic performance of the LODDS was superior to that of both the N and LNR.

Furthermore, patients in the LODDS2 or LODDS3 subgroups had poorer outcomes compared with those in the LODDS1 subgroup when stratified by the time of diagnosis, suggesting that the time of diagnosis had little effect on the prognosis of the LODDS stage in ESCC. To date, it is widely accepted that a minimum of 16 retrieved LNs are required for adequate evaluation of the LN status.^35^ According to our further results, regardless of the total number of removed LNs, the LODDS stage manifested marked superiority, particularly when the number of retrieved LNs was insufficient.

The clear tendency of a higher linear trend *χ* ^2^ score and likelihood ratio *χ* ^2^ score with the LODDS stage versus the other stages was observed in both the SEER and validation cohorts, revealing that the LODDS classification is a better predictor in prognostic prediction. Furthermore, the model incorporating the stratified LODDS stage had the highest C‐index and smallest AIC value among the three investigated nodal staging systems, implying that the LODDS stage had better discrimination ability and accuracy than the other stages in predicting survival and might be an optimal prognosis stratification system. The results of the validation cohort analysis were identical to those of the discovery cohort analysis. Interestingly, the results of subgroup analyses stratified by different characteristics in the SEER database and our cohort database indicated that the LODDS stage had an advantage in terms of its ability to distinguish heterogeneous patients within various groups.

In the current study, we also tried to construct nomograms using the N stage, LNR stage, and LODDS stage. The LODDS‐based nomograms achieved a C‐index of 0.671, which was higher than that for the N‐ and LNR‐based nomograms. The results were also validated in our cohort, indicating that the LODDS stage had a better performance than the N and LNR stages.

Despite our valuable findings, this study has several limitations that merit mentioning. First, as a retrospective study based on the SEER database and a Chinese single‐institution cohort, the clinical and pathological features may vary with different registries or hospitals, and the variables enrolled in the two cohorts were not completely consistent. Second, the SEER database has an inevitable inherent bias and was unavailable for the surgical approach, margin status, radiotherapy, chemotherapy, and some other information, which may contribute to misleading results. Third, the C‐index of the nomogram was good but it had not yet reached a high degree of distinction. The large number of patients in the population‐based database may compensate for the drawbacks resulting from the lack of information. Further studies based on multicenter or large populations with longer follow‐up times are needed to externally validate our findings more convincingly.

In conclusion, the prognostic role of LODDS and the superiority of LODDS in predicting survival compared with either the traditional N stage or LNR were confirmed in ESCC patients undergoing surgical resection. LODDS can serve as a candidate indicator to provide prognostic guidance for ESCC patients.

## CONFLICT OF INTEREST

No potential conflict of interest is disclosed.

## ETHICAL STATEMENT

This study was approved by the Ethics Committee of Tianjin Medical University Cancer Institute. The study outcomes will not affect the future management of the patients. Informed consent was obtained from the patients or their relatives.

## Supporting information

Fig S1

Fig S2

## Data Availability

The data described in the manuscript will be made available upon request.
