# Supplementary material for: The prognostic performance of the log odds of positive lymph nodes in patients with esophageal squamous cell carcinoma: A population study of the US SEER database and a Chinese single‐institution cohort
Source: Cancer Med. 2021 Jul 9;10(17):6149–64. doi: 10.1002/cam4.4120 (PMC8419772; doi:10.1002/cam4.4120)
Supplement: Supplementary file 1 — Fig S1 [file CAM4-10-6149-s001.pdf]

A

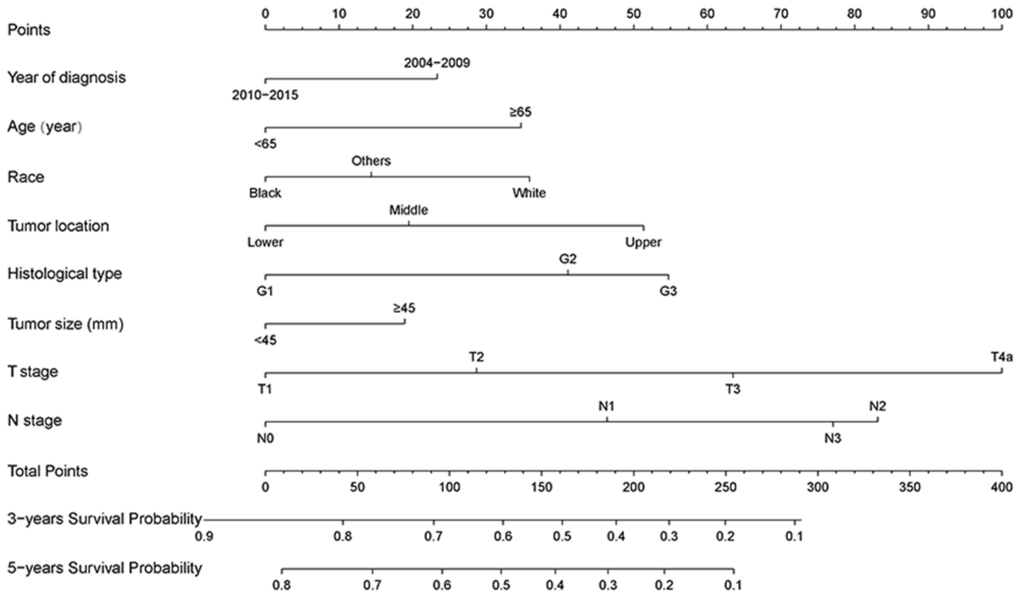

Points
0
10
20
30
40
50
60
70
80
90
100
Year of diagnosis
2004−2009
2010−2015
Age (year)
≥65
<65
Race
Others
Black
White
Tumor location
Middle
Lower
Upper
Histological type
G2
G1
G3
Tumor size (mm)
≥45
<45
T stage
T2
T4a
T1
T3
N stage
N1
N2
N0
N3
Total Points
0
50
100
150
200
250
300
350
400
3−years Survival Probability
0.9
0.8
0.7
0.6
0.5
0.4
0.3
0.2
0.1
5−years Survival Probability
0.8
0.7
0.6
0.5
0.4
0.3
0.2
0.1


B

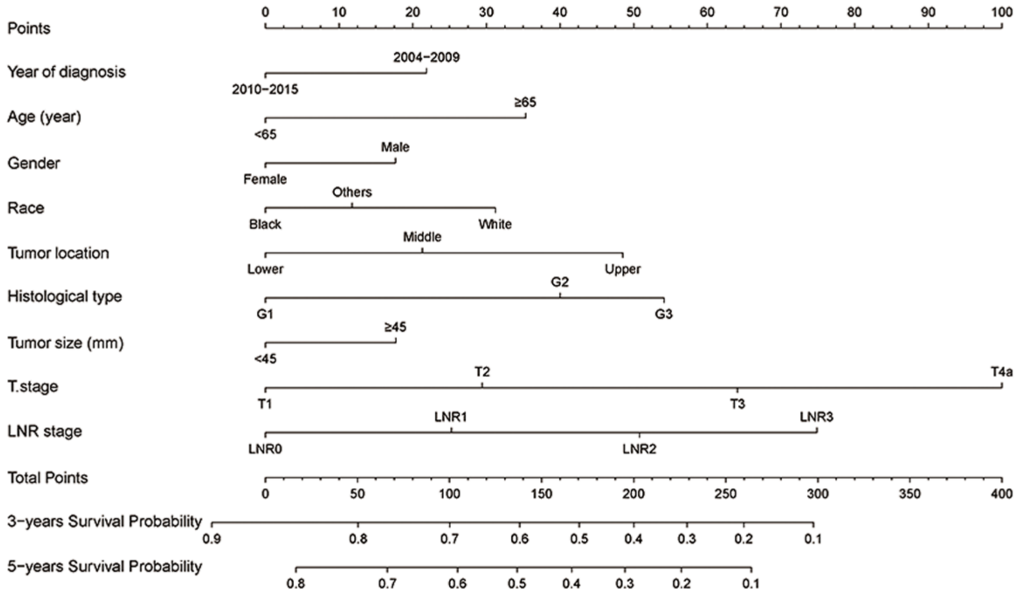

Points
0
10
20
30
40
50
60
70
80
90
100
Year of diagnosis
2004−2009
2010−2015
Age (year)
≥65
<65
Gender
Male
Female
Race
Others
Black
White
Tumor location
Middle
Lower
Upper
Histological type
G2
G1
G3
Tumor size (mm)
≥45
<45
T.stage
T2
T4a
T1
T3
LNR stage
LNR1
LNR3
LNR0
LNR2
Total Points
0
50
100
150
200
250
300
350
400
3−years Survival Probability
0.9
0.8
0.7
0.6
0.5
0.4
0.3
0.2
0.1
5−years Survival Probability
0.8
0.7
0.6
0.5
0.4
0.3
0.2
0.1

C

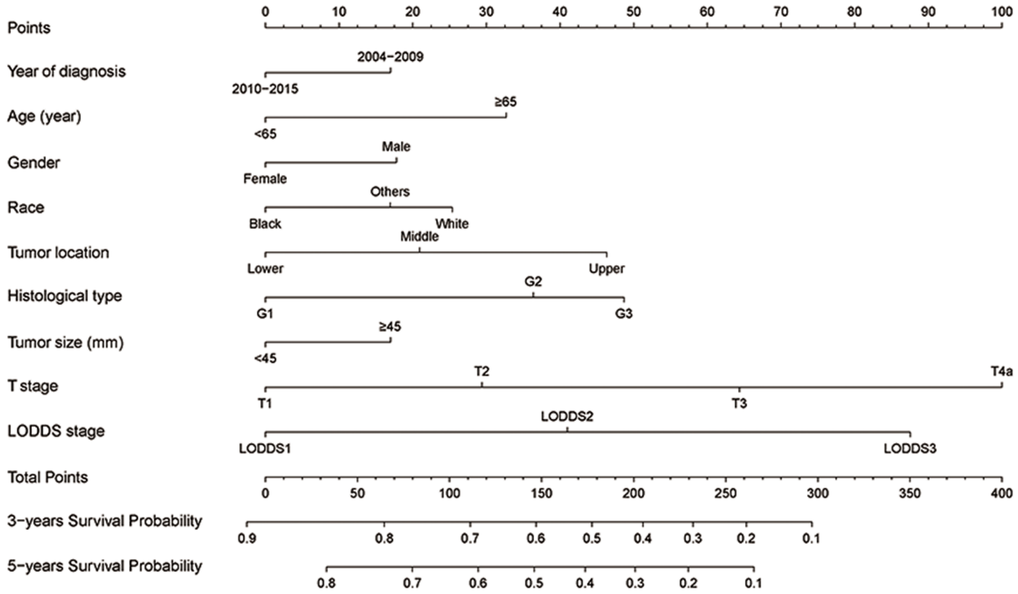


**Supplementary Figure S1**. Nomograms based on the N stage (A), LNR stage (B) and LODDS stage (C) for predicting the 3-year and 5-year OS of patients with ESCC in the SEER database.
