# Supplementary material for: The prognostic performance of the log odds of positive lymph nodes in patients with esophageal squamous cell carcinoma: A population study of the US SEER database and a Chinese single‐institution cohort
Source: Cancer Med. 2021 Jul 9;10(17):6149–64. doi: 10.1002/cam4.4120 (PMC8419772; doi:10.1002/cam4.4120)
Supplement: Supplementary file 2 — Fig S2 [file CAM4-10-6149-s002.pdf]

A

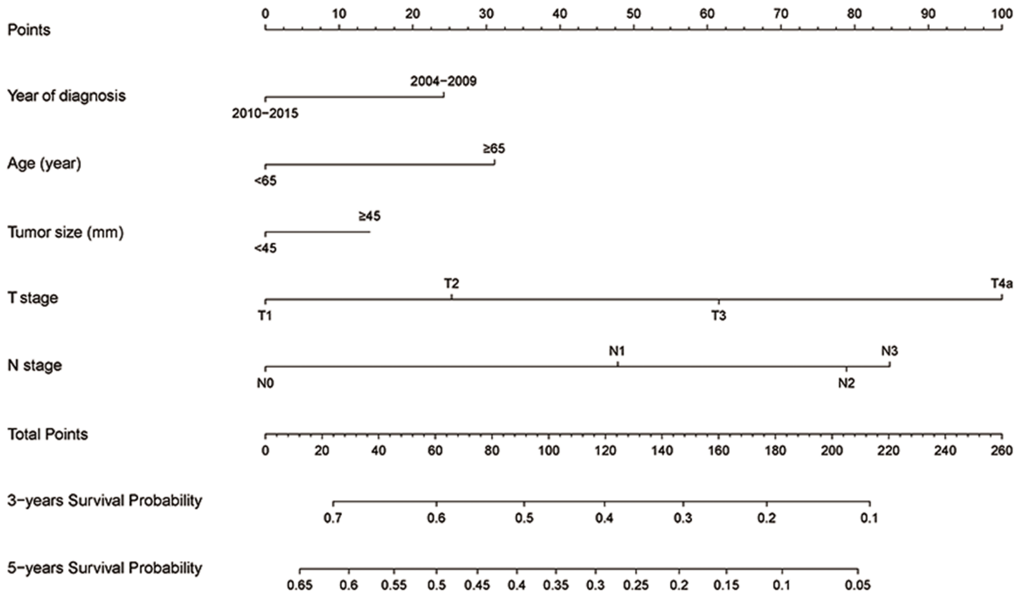
Points
0 10 20 30 40 50 60 70 80 90 100
Year of diagnosis
2004−2009
2010−2015
Age (year)
≥65
<65
Tumor size (mm)
≥45
<45
T stage
T2
T4a
T1
T3
N stage
N1
N3
N0
N2
Total Points
0 20 40 60 80 100 120 140 160 180 200 220 240 260
3−years Survival Probability
0.7 0.6 0.5 0.4 0.3 0.2 0.1
5−years Survival Probability
0.65 0.6 0.55 0.5 0.45 0.4 0.35 0.3 0.25 0.2 0.15 0.1 0.05

B

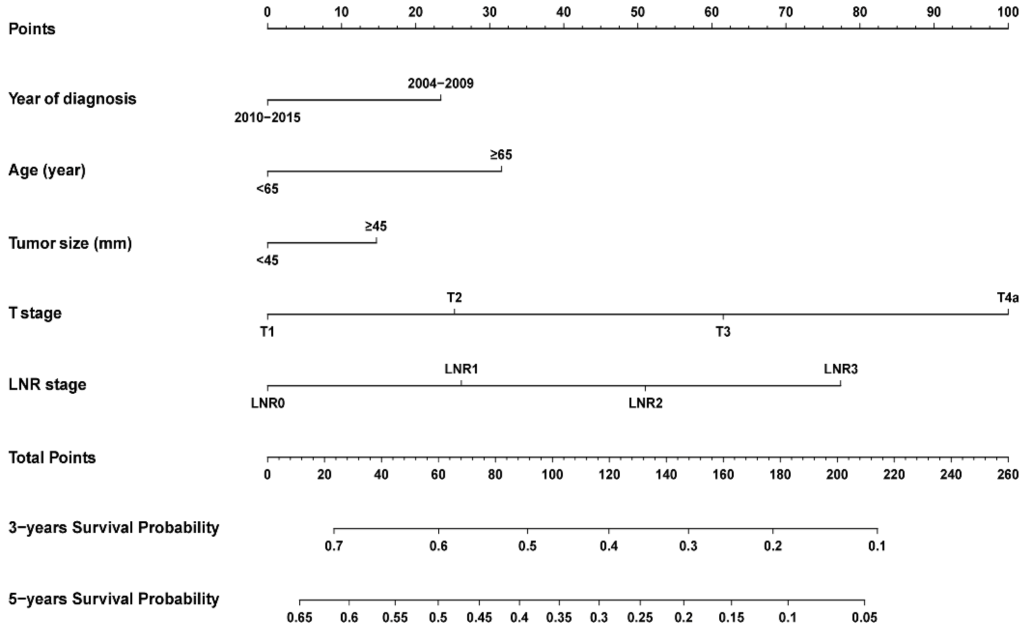
Points
0 10 20 30 40 50 60 70 80 90 100
Year of diagnosis
2004−2009
2010−2015
Age (year)
≥65
<65
Tumor size (mm)
≥45
<45
T stage
T2
T4a
T1
T3
LNR stage
LNR1
LNR3
LNR0
LNR2
Total Points
0 20 40 60 80 100 120 140 160 180 200 220 240 260
3−years Survival Probability
0.7 0.6 0.5 0.4 0.3 0.2 0.1
5−years Survival Probability
0.65 0.6 0.55 0.5 0.45 0.4 0.35 0.3 0.25 0.2 0.15 0.1 0.05

C

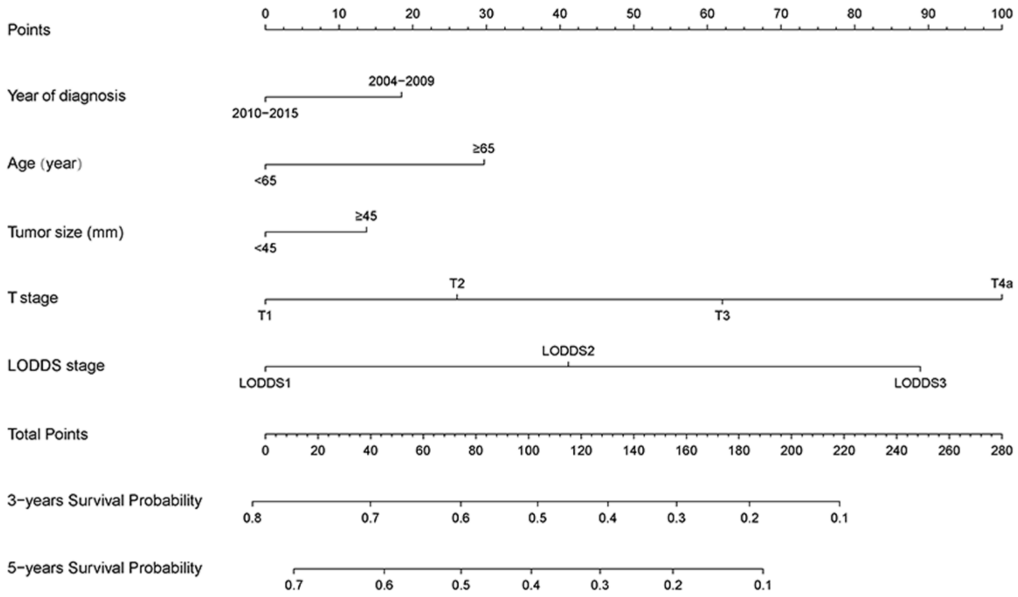

Supplementary Figure S2. Nomograms based on the N stage (A), LNR stage (B) and LODDS stage (C) for predicting the 3-year and 5-year OS of patients with ESCC in our validation cohort.
